# Supplementary material for: Epidemiology of sports-related fatalities during organized school sports in Japanese high schools between 2009 and 2018
Source: PLoS One. 2021 Aug 20;16(8):e0256383. doi: 10.1371/journal.pone.0256383 (PMC8378710; doi:10.1371/journal.pone.0256383)
Supplement: Attachment — Submitted filename: Response to Reviewers.docx [file pone.0256383.s001.docx]

Reviewers' comments:

Reviewer's Responses to Questions

**Comments to the Author**

1. Is the manuscript technically sound, and do the data support the conclusions?

The manuscript must describe a technically sound piece of scientific research with data that supports the conclusions. Experiments must have been conducted rigorously, with appropriate controls, replication, and sample sizes. The conclusions must be drawn appropriately based on the data presented.

Reviewer #1: Yes

Reviewer #2: Yes

Reviewer #3: Partly

2. Has the statistical analysis been performed appropriately and rigorously?

Reviewer #1: Yes

Reviewer #2: N/A

Reviewer #3: No

3. Have the authors made all data underlying the findings in their manuscript fully available?

The [PLOS Data policy](http://www.plosone.org/static/policies.action#sharing) requires authors to make all data underlying the findings described in their manuscript fully available without restriction, with rare exception (please refer to the Data Availability Statement in the manuscript PDF file). The data should be provided as part of the manuscript or its supporting information, or deposited to a public repository. For example, in addition to summary statistics, the data points behind means, medians and variance measures should be available. If there are restrictions on publicly sharing data—e.g. participant privacy or use of data from a third party—those must be specified.

Reviewer #1: Yes

Reviewer #2: Yes

Reviewer #3: Yes

4. Is the manuscript presented in an intelligible fashion and written in standard English?

PLOS ONE does not copyedit accepted manuscripts, so the language in submitted articles must be clear, correct, and unambiguous. Any typographical or grammatical errors should be corrected at revision, so please note any specific errors here.

Reviewer #1: Yes

Reviewer #2: No

Reviewer #3: Yes

5. Review Comments to the Author

Please use the space provided to explain your answers to the questions above. You may also include additional comments for the author, including concerns about dual publication, research ethics, or publication ethics. (Please upload your review as an attachment if it exceeds 20,000 characters)

Reviewer #1: Original article. Simple and correct statistical study. I think the accident database is very useful and interesting. I think the total number is too low to make a correlation between the number of accidents and the type of sport practiced. the data should be correlated more with the frequency of sport. overall very interesting.

**Thank you very much for your valuable feedback on our work.**

**We would like to note that in our original submission, we limited our calculation of the incidence rate of fatality in sports that reported five or more cases during the study period. Nevertheless, we acknowledge the concern raised by Reviewer 1, and have added the limitation regarding the small case number in the revised document (L339-341).**

Reviewer #2: It is a pretty good summary of sport-related fatality in Japan. The amount of this work is enough and its analyses are comprehensive which can offer quite objective data for related stakeholder.
There are also some problems in this article: 1. language problem. For example, we say 'athletic trainer' instead of 'school nurse' professionally 2.analyses problem. This article doesn't mention the specific data of medical staff which I think is valuable for continuous improvement for school.
This is my suggestion above. Thank you very much.

**Thank you very much for your valuable feedback on our work.**

**Thank you for pointing out the discrepancy regarding the nomenclature of profession. In Japan, most schools do not employ athletic trainers and they have school nurses to manage health and safety of student athletes. The use of school nurse as a term to describe the first responder was not by mistake but accurately reflects the situation in Japan.**

**Regarding the medical staff data, we were unable to obtain information about the presence of medically trained personnel in most case reports submitted to the JSC database. Therefore, our finding is limited to what is already described in the original document.**

Reviewer #3: In the manuscript by Suzuki-Yamanaka M. et al., the authors examined the frequency and incidence rate of sports-related fatalities in Japanese high schools between 2009 and 2018. Although an interesting and potentially important study, several issue should be considered.

**Thank you very much for your valuable feedback on our work. We have made changes to reflect your comments in the manuscript.**

1. The data was collected from insurance company and may be subjected to potential bias. Information on medical record or cross-validation with local or national death registry is lacking.

**We recognize the limitation of incidence rates calculation based on insurance claims. However, there currently is no additional mechanism to capture sports-related catastrophic incidents data in Japan. As almost all high schools and their students (97.7% in 2018) subscribe to the JSC Injury and Accident Mutual Aid Benefit System, and no competing companies exist for similar coverage, we believe the current data presented in our manuscript is the most reliable estimates.**

2. In the “method” part, description on data analysis is rather simple. It is unclear how the incidence rate is calculated and adjusted by what types of variables.

**Thank you for this comment. We have added details on how we carried incidence rate calculations for the subdivisions (year, sports, and type of fatality).**

3. Conclusions are not supported by statistical analysis.

**We modified the last sentence in the conclusion section to *“To ensure the health and safety of student-athletes, distribution of medically trained personnel in high school sports and improved coaching education to appropriately respond to medical emergency should be promoted in Japan”* given the finding that medically trained personnel were involved in initial care only in two cases, and the in the majority of cases, coaches were the first responder.**
