# Supplementary material for: Epidemiology of sports-related fatalities during organized school sports in Japanese high schools between 2009 and 2018
Source: PLoS One. 2021 Aug 20;16(8):e0256383. doi: 10.1371/journal.pone.0256383 (PMC8378710; doi:10.1371/journal.pone.0256383)
Supplement: Attachment — Submitted filename: Response to Reviewers.docx [file pone.0256383.s002.docx]

Reviewer's Responses to Questions

Reviewer #3: The manuscript has been modified to incorporate most of my suggestions. It seems that the "Conclusion" of the manuscript could still be specified and refined. Currently, the conclusion is a compilation of numbers and "jump" to a wide statement (the last sentence). The readers may benefit from more detailed conclusions like "fatality by indirect causes such cardiac failure dropped greatly due to XX policy" or "The high fatality rate in Judo was mainly caused by direct causes such as collisons that could be reduced by implement XX policy", rather than simply list the numbers.

**Thank you very much for providing valuable feedback. We incorporated a couple of sentences in the conclusion section to better explain the findings of this study.**
